# Characterization of the developing small intestine in the absence of either GATA4 or GATA6

**DOI:** 10.1186/1756-0500-7-902

**Published:** 2014-12-11

**Authors:** Emily M Walker, Cayla A Thompson, Bridget M Kohlnhofer, Mary L Faber, Michele A Battle

**Affiliations:** Department of Cell Biology, Neurobiology and Anatomy, Medical College of Wisconsin, 8701 Watertown Plank Road, Milwaukee, WI 53226 USA

**Keywords:** GATA4, GATA6, Small intestine, Development, Epithelium, Conditional knockout

## Abstract

**Background:**

Studies of adult mice lacking either GATA4 or GATA6 in the small intestine demonstrate roles for these factors in small intestinal biology. Deletion of *Gata4* in the adult mouse intestine revealed an essential role for GATA4 in jejunal function. Deletion of *Gata6* in the adult mouse ileum alters epithelial cell types and ileal enterocyte gene expression. The effect of deletion of *Gata4* or *Gata6* alone during embryonic small intestinal development, however, has not been examined. We recently demonstrated that loss of both factors in double conditional knockout embryos causes severe defects in jejunal development. Therefore, the goal of this study is to provide phenotypic analysis of the small intestine of single *Gata4* and *Gata6* conditional knockout embryos.

**Results:**

*Villin-Cre* was used to delete *Gata4* or *Gata6* in the developing intestinal epithelium. Elimination of either GATA4 or GATA6 in the jejunum, where these factors are co-expressed, caused changes in enterocyte and enteroendocrine cell gene expression. Ectopic expression of markers of the ileal-specific bile acid metabolism pathway was induced in GATA4-deficient jejunum but not in GATA6-deficient jejunum. A subtle increase in goblet cells was also identified in jejunum of both mutants. In GATA6-deficient embryonic ileum, villus length was altered, and enterocyte gene expression was perturbed including ectopic expression of the colon marker *Car1*. Goblet cells were increased, and enteroendocrine cells were decreased.

**Conclusions:**

Overall, we show that aspects of the phenotypes observed in the small intestine of adult *Gata4* and *Gata6* conditional knockout mice emerge during development. The effect of eliminating GATA6 from the developing ileum was greater than that of eliminating either GATA4 or GATA6 from the developing jejunum likely reflecting functional redundancy between these factors in the jejunum. Although GATA4 and GATA6 functions overlap, our data also suggest unique functions for GATA4 and GATA6 within the developing intestine. GATA4 likely operates independently of GATA6 within the jejunum to regulate jejunal versus ileal enterocyte identity and consequently jejunal physiology. GATA6 likely regulates enteroendocrine cell differentiation cell autonomously whereas GATA4 affects this population indirectly.

**Electronic supplementary material:**

The online version of this article (doi:10.1186/1756-0500-7-902) contains supplementary material, which is available to authorized users.

## Background

The zinc-finger DNA binding transcription factors GATA4 and GATA6 are expressed in the small intestinal epithelium throughout development and adulthood [1–7]. GATA4, unlike GATA6, is present in a restricted pattern in the adult small intestine; it is expressed in the proximal small intestine (duodenum and jejunum) but absent from the distal small intestine (ileum) [1, 4, 8]. Global *Gata4* or *Gata6* knockout causes early embryonic lethality necessitating conditional knockout (cKO) strategies to analyze their roles in organogenesis [5, 9–11]. Several studies performed by our laboratory and others to eliminate GATA4 or GATA6 specifically in the intestinal epithelium have uncovered roles for these factors in small intestinal biology [1, 2, 4]. Fat and cholesterol absorption are disrupted in adult mice lacking GATA4 in the jejunal epithelium [1]. Moreover, expression of many jejunal-specific transcripts is lost and expression of many ileal-specific transcripts is induced in GATA4-deficient jejunum demonstrating a role for GATA4 in regulating jejunal versus ileal intestinal identity [1, 4]. Although constitutive *Villin-Cre* has been used to delete *Gata4* in the small intestine during development [1], GATA4-deficient embryonic intestine was not examined.

Unlike *Gata4* cKO adult mice, elimination of *Gata6* from the adult jejunal epithelium using tamoxifen-inducible *Villin-Cre* does not decrease expression of jejunal enterocyte markers or induce expression of ileal enterocyte markers suggesting that jejunal identity is maintained in its absence [2]. Increased Paneth cells with atypical granules are reported in GATA6-deficient jejunum [2]. In contrast, loss of *Gata6* from the adult ileum, a tissue lacking GATA4, results in shortened villi, reduced proliferative, enteroendocrine, and Paneth cells, and increased crypt goblet cells [2]. Changes in ileal enterocyte gene expression also occur; small intestinal enterocyte marker expression is decreased, and colonocyte marker expression is induced suggesting that GATA6 plays a role in regulating intestinal identity in the distal small intestine [2]. These studies did not induce *Gata6* deletion during embryonic development precluding analysis of embryonic intestine.

We recently demonstrated that simultaneous deletion of both *Gata4* and *Gata6* within the developing intestinal epithelium using constitutive *Villin-Cre* severely disrupts jejunal development causing *Gata4-Gata6* double cKO mice to die within a day of birth [12]. Intestinal epithelial architecture is altered in the absence of both GATA4 and GATA6 with the jejunum of *Gata4-Gata6* double cKO embryos containing short, blunted villi. Furthermore, differentiated epithelial cell populations are skewed in *Gata4-Gata6* double cKOs. Enterocytes are decreased and goblet and proliferative cells are increased in mutant jejunum. The effect of deletion of *Gata4* or *Gata6* alone during embryonic development of the small intestine, however, has not been examined. Therefore, the goal of this study is to provide phenotypic analysis of intestinal development in single *Gata4* and *Gata6* cKO embryos derived using constitutive *Villin-Cre*. We examined the jejunum of *Gata4 Villin-Cre* and *Gata6 Villin-Cre* cKO embryos and the ileum of *Gata6 Villin-Cre* cKO embryos at E18.5. We found that jejunum lacking either GATA4 or GATA6 was largely normal. Changes in enterocyte gene expression reflecting a transition from jejunal to ileal identity were identified only in GATA4 mutant jejunum. Analysis of GATA6 mutant ileum revealed a phenotype similar to that observed when GATA6 is eliminated from the adult ileal epithelium. We observed shortened villi and subtle changes in epithelial cell populations including altered enterocyte gene expression, decreased enteroendocrine cells, and increased goblet and proliferative cells.

## Results and discussion

**Figure 1:**
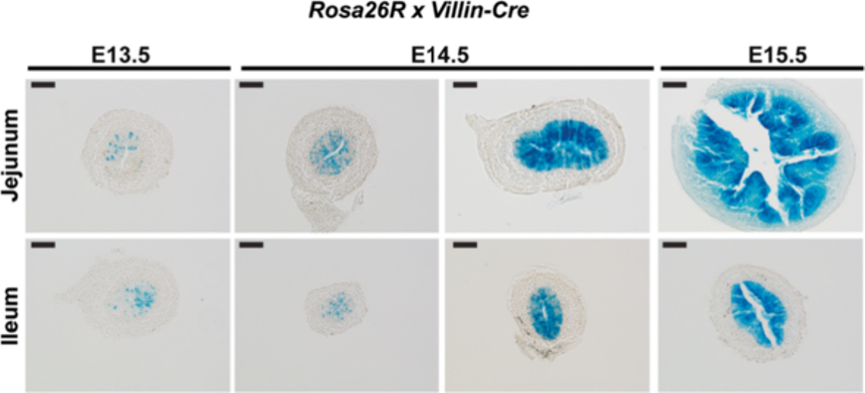
**Villin-Cre shows maximal activity between E14.5-E15.5.** *Villin-Cre* (Tg(*Vil-cre*)997Gum) and *Rosa26R* mice (*Gt(ROSA)26Sor* ^*tm1Sor*^) were crossed to determine Cre activity. Embryonic small intestine harvested at E13.5, E14.5, and E15.5 was stained for β-galactosidase activity (blue staining) to identify Cre activity. At E13.5, minimal Cre activity was detected in jejunum and ileum (n = 9 Cre + embryos from 2 litters; 5/9 showed scattered blue cells; 4/9 had no blue cells). At E14.5, two types of staining were detected (n = 11 Cre + embryos from 2 litters). One group (4/11) had heterogeneous staining in jejunum and ileum (E14.5 left panel). The other group (7/11) had robust homogeneous staining in jejunum and ileum (E14.5 right panel). At E15.5, we observed 3/4 Cre + embryos with robust homogeneous staining in jejunum and ileum. One Cre + embryo had a low level of blue staining reminiscent of earlier stages (n = 4 Cre + embryos from 1 litter). Scale bars, 50 μm.

To determine GATA4 or GATA6 function during small intestinal development, we generated intestinal epithelium-specific *Gata4* and *Gata6* cKO embryos using the following mouse lines: *Gata4*^*tm1.1Sad*^*(Gata4*^*loxP*^), *Gata4*^*tmo1Eno*^ (*Gata4*^*−*^), *Gata6*^*tm2.1Sad*^ (*Gata6*^*loxP*^), *Gata6*^*tm2.2Sad*^ (*Gata6*^*−*^), and *Tg(Vil-cre)997Gum (Villin-Cre*) [10, 13–15]. For our studies, control embryo genotypes were *Gata4*^*loxP/+*^ or *Gata6*^*loxP/+*^ and experimental embryo genotypes were *Gata4*^*loxP/-*^*Villin-Cre* or *Gata6*^*loxP/-*^*Villin-Cre.* Although *Villin-Cre* is widely used to generate genetic deletions within the intestinal epithelium, the time course of Villin-Cre activity within the embryonic small intestinal epithelium has not been shown. Therefore, we mated *Villin-Cre* mice with the *Rosa26* conditional LacZ reporter strain *Gt(ROSA)26Sor*^*tm1Sor*^*(Rosa26R)*[16] and stained embryonic intestine for β-galactosidase activity between E13.5-E15.5. We found minimal Cre activity present at E13.5 (Figure 1). By E14.5, the intestinal epithelium of most embryos analyzed (7/11) showed robust Cre activity (Figure 1). A small subset of E14.5 embryos (4/11) had heterogeneous intestinal Cre activity (Figure 1). By E15.5, the majority of embryos examined (3/4) showed robust intestinal Cre activity (Figure 1). Therefore, we conclude that the *Villin* promoter drives robust Cre recombinase activity in the small intestinal epithelium by E14.5-15.5 (Figure 1). The heterogeneity in Cre activity we observed at E14.5 and E15.5 could reflect differences in Cre activity itself or in the developmental maturity of embryos within litters.

**Figure 2:**
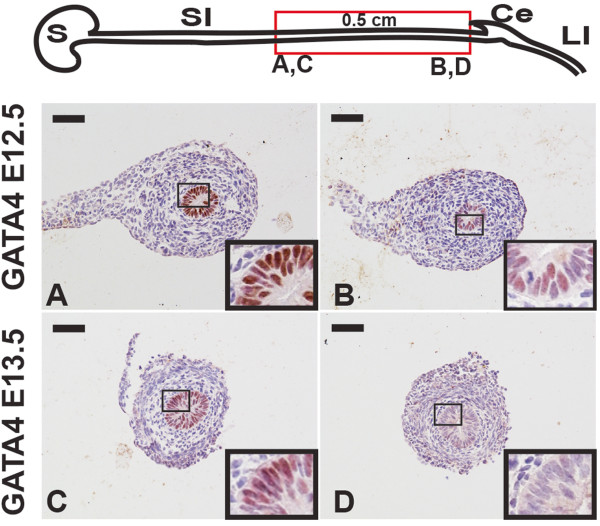
**GATA4 is excluded from the distal small intestinal epithelium during early development.** Immunohistochemistry (IHC) for GATA4 (brown nuclear stain) is shown in tissue from the distal region of small intestine of E12.5 (n = 3) and E13.5 (n = 7) CD1 embryos. A 0.5 cm piece of small intestine dissected just superior to the cecum (red box) from each embryo was sectioned through its entirety and stained for GATA4. Tissue was also counterstained with hematoxylin. GATA4 was present throughout the region examined at E12.5 (panels **A** and **B**) although scattered cells within the most distal section of the tissue (panel **B**) had already lost GATA4 expression. By E13.5, GATA4 protein was absent from all cells in the most distal region of the small intestine (compare panels **C** and **D**). Insets show higher magnification images of boxed regions. S, stomach; SI, small intestine; Ce, cecum; LI, large intestine. Scale bars, 50 μm.

**Figure 3:**
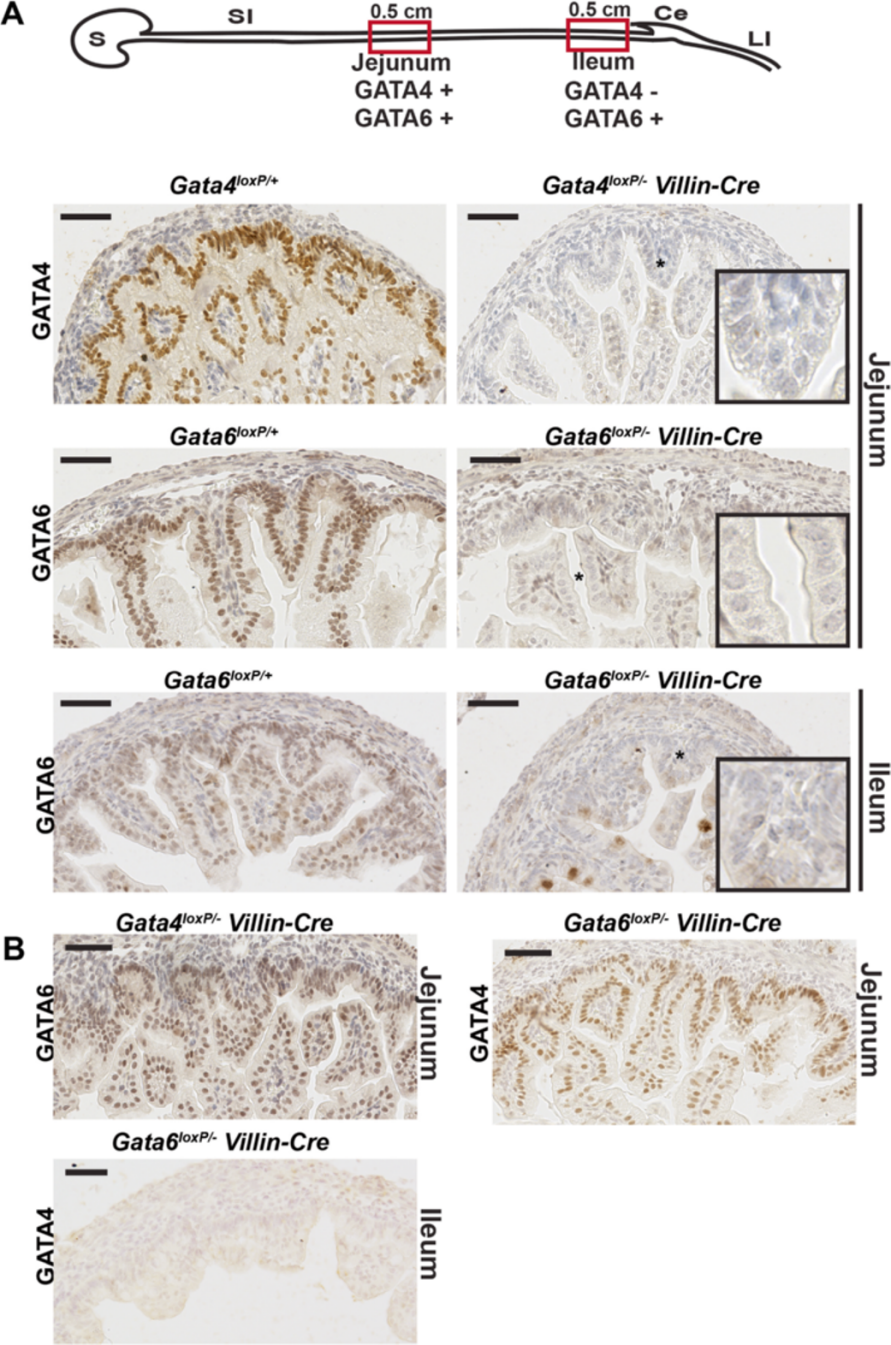
**GATA4 or GATA6 were eliminated from the intestinal epithelium using** ***Villin-Cre.*** **(A)** IHC for either GATA4 or GATA6 (brown nuclear stain) demonstrated that GATA4 and GATA6 protein were eliminated from the intestinal epithelium of mutants at E18.5. Inset shows higher magnification image of starred region. Diagram shows the regions from which tissue was harvested for IHC (red boxes). S, stomach; SI, small intestine; Ce, cecum; LI, large intestine. **(B)** IHC for GATA6 in GATA4 mutant jejunum confirmed that GATA6 protein was maintained in the absence of GATA4. IHC for GATA4 in GATA6 mutant jejunum confirmed that GATA4 protein was maintained in the absence of GATA6. IHC for GATA4 in GATA6 mutant ileum demonstrated that GATA4 was not ectopically induced in the absence of GATA6. Scale bars, 50 μm. All experiments performed using E18.5 embryos.

Although GATA4 is absent from the distal small intestine of late stage embryos (E17.5) and adult mice [1, 3, 4, 8], the time at which restricted intestinal GATA4 expression is first detectable is unknown. The relationship between GATA4 restriction and onset of Villin-Cre activity is relevant to our study to identify which regions of the gut will be affected by *Gata4* deletion. Therefore, to determine the earliest time at which GATA4 expression is restricted, we isolated the distal most 0.5 cm of mouse embryonic small intestinal tissue at various gestational ages and used immunohistochemistry to identify GATA4. At E12.5, GATA4 positive cells were found throughout the distal small intestine (Figure 2A, B). Although most cells within this region expressed GATA4, we did identify some GATA4 negative cells (Figure 2B). By E13.5, we observed loss of GATA4 from all cells of the most distal region of the small intestine (Figure 2C, D). Therefore, we conclude that GATA4 is initially expressed throughout the small intestinal epithelium and that it becomes excluded from the distal small intestinal epithelium at E12.5-E13.5. Moreover, because Villin-Cre did not become maximally activated until E14.5-E15.5, we conclude that *Gata4* knockout using Villin-Cre should not impact development of the distal small intestine (ileum) as GATA4 is lost in this region prior to Cre activity. Therefore, we limited our analysis of *Gata4* cKO embryos to jejunum. Because GATA6 is expressed throughout the small intestinal epithelium [2, 8], we analyzed both jejunum and ileum of *Gata6* cKO embryos. We validated loss of GATA4 or GATA6 protein in small intestine from E18.5 *Gata4* cKO and *Gata6* cKO embryos using immunohistochemistry (Figure 3A). Moreover, we examined tissue lacking GATA4 or GATA6 to determine if expression of the remaining GATA factor changed. We found that GATA4 was maintained in GATA6-deficient jejunum and that GATA6 was maintained in GATA4-deficient jejunum (Figure 3B). Finally, GATA4 was not induced in GATA6-deficient ileum (Figure 3B). Analysis of *Gata4* and *Gata6* mRNA levels by qRT-PCR confirmed these results (Additional file 1).

**Figure 4:**
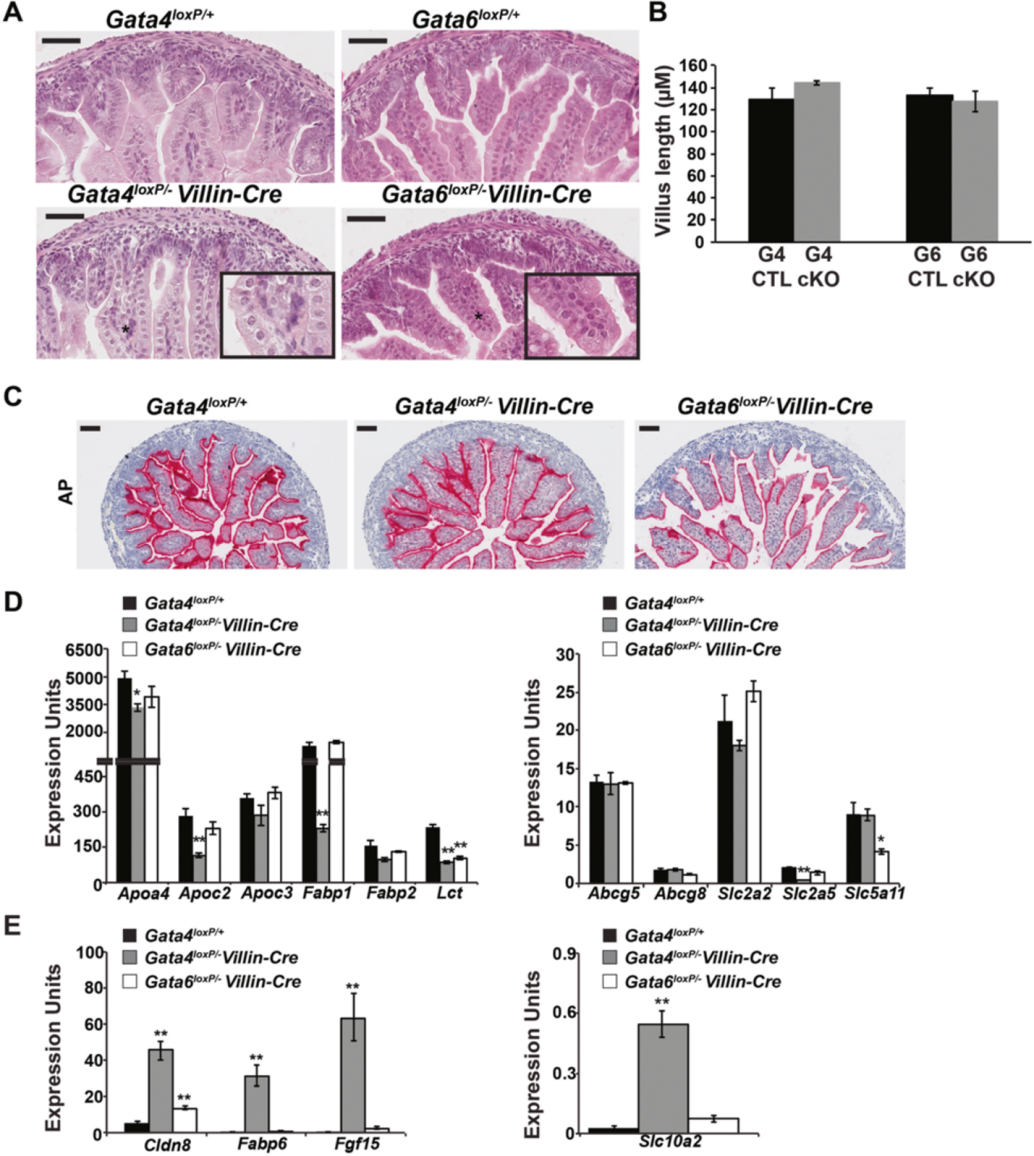
**GATA4 or GATA6 deletion in the jejunal epithelium perturbs enterocyte gene expression. (A)** Hematoxylin and eosin (H&E) staining of jejunal tissue from controls and GATA4 or GATA6 mutants showed no difference in intestinal architecture. **(B)** Average jejunal villus length was determined using NDP Scan software (Hamamatsu) to measure the length of all villi per section. No difference was observed between controls and GATA4 or GATA6 mutants (G4 CTL, *Gata4* ^*loxP/+*^, n = 4 embryos, 3 sections/embryo, 166 villi, 129.2 ± 10.8 μm; G4 cKO, *Gata4* ^*loxP/-*^ *Villin-Cre*, n = 3 embryos, 3 sections/ embryo, 132 villi, 144.1 ± 1.8 μm; G6 CTL, *Gata6* ^*loxP/+*^, n = 4 embryos, 3 sections/ embryo, 189 villi, 132.7 ± 6.6 μm; G6 cKO, *Gata6* ^*loxP/-*^ *Villin-Cre*, n = 4 embryos, 3 sections/embryo, 173 villi, 127.7 ± 9.4 μm. Differences are not statistically significant) **(C)** Alkaline phosphatase (AP) staining was equivalent between controls and mutants. **(D)** qRT-PCR for jejunal-enriched enterocyte transcripts (*Apoa4, Apoc2*, *Apoc3, Fabp1, Lct, Slc2a2, Slc2a5, Slc5a11)* showed that 5/8 were decreased in GATA4 mutants and that 2/8 were decreased in GATA6 mutants. None of the transcripts expressed at similar levels in jejunum and ileum (*Fabp2, Abcg5, Abcg8*) were changed in either mutant compared with controls. **(E)** qRT-PCR for ileal-enriched enterocyte transcripts (*Slc10a2, Fabp6, Fgf15*, *Cldn8*) showed that expression of each was induced in jejunum in the absence of GATA4. Only *Cldn8* was induced in jejunum in the absence of GATA6. For qRT-PCR in D and E, epithelial cells from three control (*Gata4* ^*loxP/+*^) and three mutant intestines (*Gata4* ^*loxP/-*^ *Villin-Cre* or *Gata6* ^*loxP/-*^ *Villin-Cre)* were assayed at least three times. *Gapdh* was used for normalization. All *P*-values were determined by two-sample Student’s *t* test: *P ≤ 0.05, **P ≤ 0.01. All error bars show SEM. Scale bars in all micrographs are 50 μm. All experiments performed using E18.5 embryos.

To identify the impact of loss of either GATA4 or GATA6 on jejunal epithelial development, we examined jejunum of *Gata4* and *Gata6* cKO embryos at E18.5. We chose to analyze GATA-deficient intestine at E18.5 because developmental processes such as villus morphogenesis and epithelial cytodifferentiation are essentially complete by this time [17], and our goal is to determine the extent to which either GATA4 or GATA6 are required to complete these processes. We recognize, however, that because the onset of Villin-Cre activity occurs earlier than E18.5, the effects that we observe in GATA mutants may represent both primary and secondary consequences of *Gata4* or *Gata6* deletion. To examine epithelial architecture in jejunum lacking GATA4 or GATA6, we used hematoxylin and eosin (H&E) staining and found GATA mutants to be indistinguishable from controls (Figure 4A). Because we have previously demonstrated that jejunal villus length is significantly decreased in adult *Gata4* cKO animals [1], we determined average villus length in control and mutant jejunal tissue. We found no difference in jejunal villus length among *Gata4* cKO, *Gata6* cKO, and control embryos (Figure 4B). Therefore, we conclude that GATA4 exerts its effect on jejunal villus length after birth. In the mouse, small intestinal development continues during the postnatal period with substantial remodeling of the mucosa occurring including replacement of intervillus regions by crypts [17]. It is possible that GATA4 acts during these processes to effect final villus length.

We further characterized specific intestinal epithelial cell types in GATA4 and GATA6 mutant jejunum using histochemistry and immunohistochemistry. To assess the enterocyte population, we stained for brush border alkaline phosphatase (AP) activity and found no difference between controls and GATA4 or GATA6 mutants (Figure 4C) suggesting that loss of GATA4 or GATA6 does not alter jejunal enterocyte number.

Because the enterocyte gene expression profile of GATA4 mutant adult jejunum shifts from that of the jejunum toward that of the ileum [1], we performed qRT-PCR using epithelial cells from control and GATA4-deficient jejunum to compare expression of sets of jejunal-enriched transcripts (*Apoa4, Apoc2*, *Apoc3, Fabp1, Lct, Slc2a2, Slc2a5,* and *Slc5a11),* ileal-enriched transcripts (*Slc10a2, Fabp6, Fgf15*, and *Cldn8*), and transcripts expressed at similar levels in jejunum and ileum (*Fabp2, Abcg5,* and *Abcg8*). The majority of jejunal-enriched transcripts examined (5/8) were decreased in GATA4 mutant epithelium compared with control (Figure 4D). Moreover, expression of all ileal-enriched transcripts examined (4/4) was increased in GATA4 mutant epithelium compared with control (Figure 4E). Notably, ectopic expression of bile acid metabolism markers (*Slc10a2*, *Fabp6*, and *Fgf15*) was induced. None of the transcripts expressed ubiquitously in jejunum and ileum were changed in GATA4 mutant epithelium (Figure 4D). Taken together, these data suggest that gene expression changes in GATA4 mutant jejunal epithelium reflect a shift in jejunal enterocyte identity toward that of the ileum rather than a change in enterocyte cell number.

In contrast to GATA4-deficient jejunum, loss of GATA6 in adult mouse jejunum fails to alter enterocyte gene expression patterns [2]. We performed qRT-PCR for the same set of transcripts examined in GATA4 mutants using jejunal epithelial cells from control and GATA6-deficient jejunum. In general, gene expression profiles were conserved in jejunal epithelium lacking GATA6. However, we did find that expression of two jejunal-enriched transcripts, *Lct* and *Slc5a11*, and one ileal-enriched transcript, *Cldn8*, was altered in GATA6 mutant epithelium (Figures 4D, E). These data suggest that loss of GATA6 during intestinal development subtly alters the jejunal enterocyte gene expression profile. Unlike GATA4-deficient jejunum, bile acid metabolism markers were not induced in jejunum lacking GATA6 suggesting that GATA4 uniquely regulates these genes in the developing jejunum.

**Figure 5:**
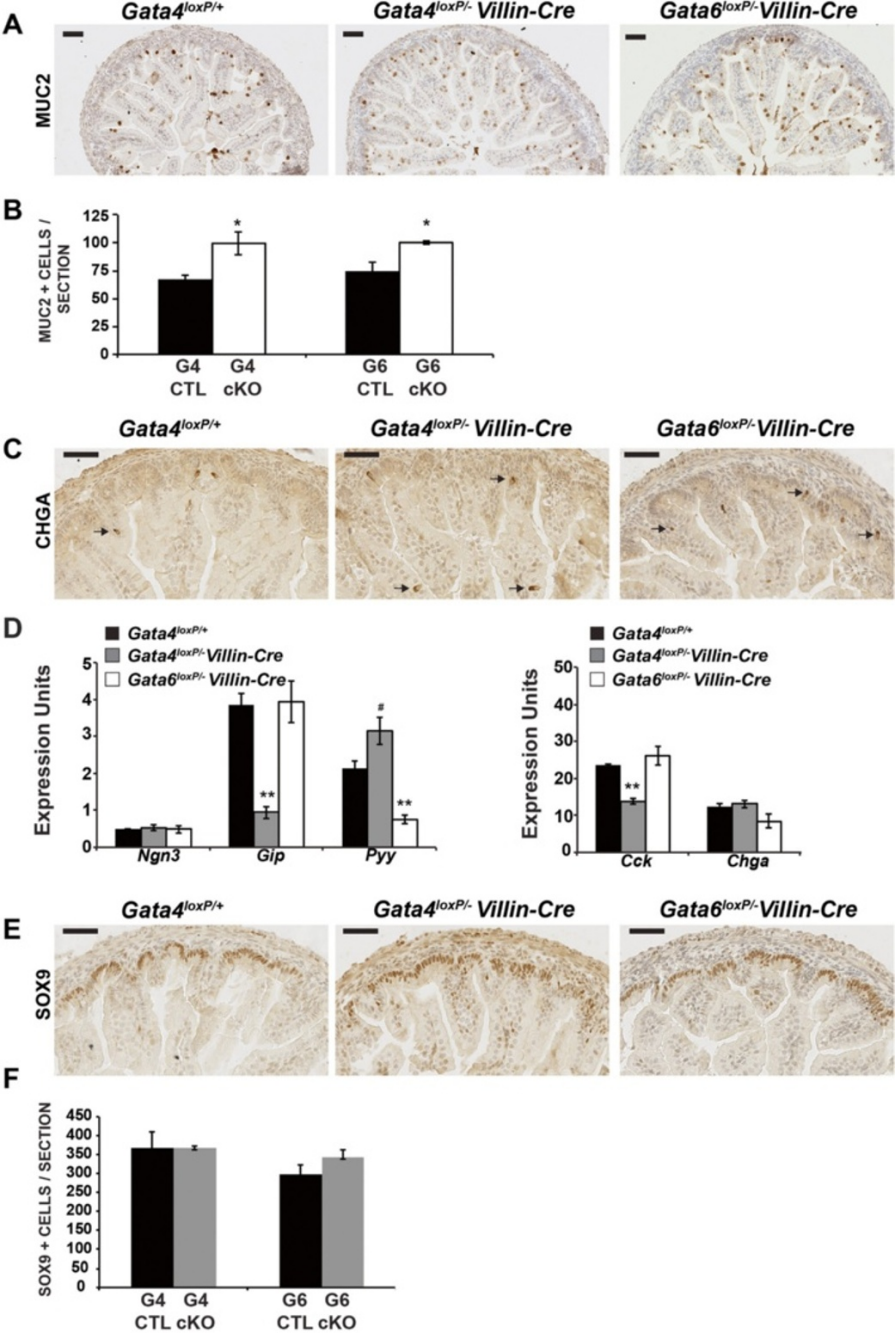
**GATA4 or GATA6 deletion in the jejunal epithelium alters goblet cell number and enteroendocrine cell gene expression. (A)** MUCIN 2 (MUC2) IHC indicated a subtle increase in goblet cells in *Gata4* and *Gata6* cKO jejunum compared with controls. **(B)** Jejunum from *Gata4* cKO and *Gata6* cKO embryos contained more goblet cells than control jejunum (G4 CTL, *Gata4* ^*loxP/+*^, 68 ± 4 MUC2+ cells/section; G4 cKO, *Gata4* ^*loxP/-*^ *Villin-Cre*, 100 ± 10 MUC2+ cells/section; G6 CTL, *Gata6* ^*loxP/+*^, 75 ± 8 MUC2+ cells/section; G6 cKO, *Gata6* ^*loxP/-*^ *Villin-Cre*, 100 ± 1 MUC2+ cells/section; n = 3 embryos/genotype, 3 sections/embryo) **(C)** Chromogranin A (CHGA) IHC appeared comparable among jejunum from *Gata4* cKO, *Gata6* cKO, and control embryos. **(D)** qRT-PCR for enteroendocrine cell markers showed that pan-enteroendocrine cell marker expression (*Ngn3, ChgA*) was unchanged in the absence of GATA4 or GATA6. Expression of proximal enteroendocrine cell markers was decreased in GATA4 mutant jejunal epithelium (*Gip, Cck*). Expression of *Pyy*, a distally-enriched enteroendocrine cell marker, was increased in GATA4 mutants and decreased in GATA6 mutants. Epithelial cells from three control (*Gata4* ^*loxP/+*^) and three mutant intestines (*Gata4* ^*loxP/-*^ *Villin-Cre* or *Gata6* ^*loxP/-*^ *Villin-Cre)* were assayed at least three times. *Gapdh* was used for normalization. **(E)** SOX9 IHC was unchanged between knockouts and controls. **(F)** Loss of GATA4 or GATA6 did not alter SOX9+ cell number in the jejunum (G4 CTL, *Gata4* ^*loxP/+*^, 367 ± 44 SOX9 + cells/section; G4 cKO, *Gata4* ^*loxP/-*^ *Villin-Cre,* 367 ± 5 SOX9 + cells/section; G6 CTL, *Gata6* ^*loxP/+*^, 299 ± 25 SOX9 + cells/section; G6 cKO, *Gata6* ^*loxP/-*^ *Villin-Cre*, 343 ± 21 SOX9 + cells/section; n = 3 embryos/genotype, 3 sections/embryo; Differences were not statistically significant). *P*-values were determined by two-sample Student’s *t* test: *P ≤ 0.05, **P ≤ 0.01, ^#^ *P* = 0.0744. Error bars show SEM. Scale bars are 50 μm. All experiments performed using E18.5 embryos.

To examine secretory cells in mutant jejunum, we stained for the goblet cell marker Mucin 2 (MUC2) and the pan-enteroendocrine cell marker Chromogranin A (CHGA). MUC2 staining revealed a 1.5-fold increase in goblet cells in GATA4-deficient jejunum and a 1.3-fold increase in goblet cells in GATA6-deficient jejunum compared with controls (Figure 5A,B). This agrees with our previously published observation that alcian blue positive goblet cells increase in the jejunum of *Gata4* and *Gata6* cKO embryos [12]. CHGA staining demonstrated a similar distribution of enteroendocrine cells within control and GATA4 or GATA6 mutant jejunum (Figure 5C). Because enteroendocrine cells constitute approximately 1% of the intestinal epithelium, we estimated enteroendocrine cell number using qRT-PCR to determine abundance of *ChgA* and *Ngn3*. We found no change in the level of *ChgA* or *Ngn3* transcripts (Figure 5D) suggesting that there is no alteration in enteroendocrine cell number GATA4 or GATA6 mutant jejunum. Examination of markers for specific enteroendocrine cell subpopulations, however, showed changes in enteroendocrine cell types present in GATA4 and GATA6 mutant jejunum (Figure 5D). GATA4-deficient jejunal epithelium contained lower levels of *Cck* and *Gip* transcripts, which are markers of proximal intestine enteroendocrine cells. Abundance of *Pyy* transcript, which is a marker of distal intestine enteroendocrine cells, increased although this change was just outside of statistical significance (P = 0.0744). Expression of these transcripts is similarly altered in the jejunum of adult *Gata4* cKO mice [1]. Because GATA4 is not expressed in enteroendocrine cells [4], changes in enteroendocrine cell subtypes in GATA4-deficient jejunum likely reflect an indirect effect of GATA4. In contrast to adult GATA6-deficient jejunum, in which no change in enteroendocrine cell marker expression occurs [2], *Pyy* transcript abundance was decreased in jejunum of E18.5 *Gata6* cKOs. Co-staining of tissue with antibodies to GATA6 and CHGA suggests that enteroendocrine cells express GATA6 (Additional file 2). Therefore, unlike GATA4 mutants, changes in abundance of enteroendocrine cell markers in GATA6 mutants may represent a cell autonomous phenotype. Moreover, it is interesting that loss of GATA4 increased *Pyy* expression whereas loss of GATA6 decreased *Pyy* expression. This difference may reflect direct regulation of *Pyy* transcription by GATA6 and indirect, non-cell autonomous regulation of *Pyy* by GATA4. Finally, we examined the intervillus region in jejunum of controls and GATA4 or GATA6 mutants by staining for the intervillus marker SOX9 and by counting SOX9+ cells. We found no difference in the localization or number of SOX9+ cells between groups suggesting that the intervillus region is normal in the absence of either GATA4 or GATA6 (Figure 5E,F).

**Figure 6:**
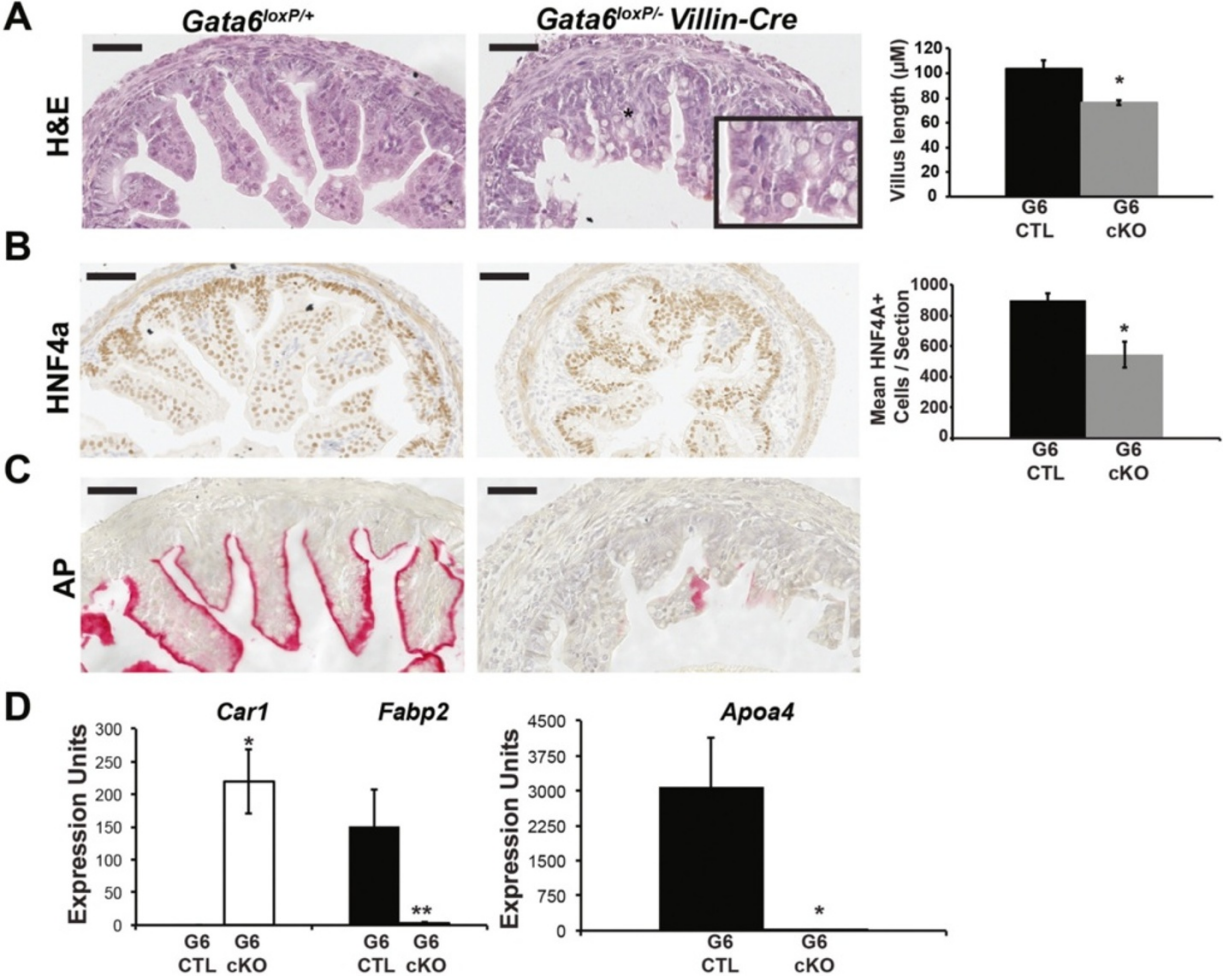
**Loss of GATA6 from the ileum disrupts the intestinal epithelial structure and enterocyte gene expression. (A)** H&E staining of ileum from control and *Gata6* cKO embryos showed that mutant villi appeared shorter than those of controls. Average ileal villus length in control and GATA6 mutant ileum was determined using NDP Scan software (Hamamatsu) to measure the length of all villi per section (G6 CTL, *Gata6* ^*loxP/+*^,117 villi,103.8 ± 7.0 μm; G6 cKO, *Gata6* ^*loxP/-*^ *Villin-Cre*, 88 villi, 76.4 ± 2.0 μm; n = 3 embryos/genotype, 3 sections/embryo). Villi were 26% shorter in GATA6 mutants compared with controls. **(B)** Control and GATA6-deficient ileum were stained for HNF4A and the number of HNF4A+ epithelial cells per section was determined. We found reduced HNF4A+ cells in GATA6 mutant ileum compared with controls (G6 CTL, *Gata6* ^*loxP/+*^, 899 ± 43 HNF4A+ cells/section; G6 cKO, *Gata6* ^*loxP/-*^ *Villin-Cre*, 543 ± 85 HNF4A+ cells/section; n = 3 embryos/genotype, 3 sections/embryo). **(C)** Alkaline phosphatase (AP) staining was reduced or absent in GATA6 mutant ileum compared with control ileum. **(D)** qRT-PCR was used to determine the abundance of *Apoa4* and *Fabp2,* small intestinal enterocyte markers, and *Car1*, a marker of colonocytes, in the ileum of control and GATA6 mutants. Both *Apoa4* and *Fabp2* levels were severely decreased in GATA6 mutant ileum compared with control ileum. *Car1* transcript was induced in GATA6 mutant ileum compared with control ileum. Epithelial cells from three control (*Gata6* ^*loxP/+*^ *)* and four mutant ileums (*Gata6* ^*loxP/-*^ *Villin-Cre)* were assayed at least three times. *Gapdh* was used for normalization. All *P*-values were determined by two-sample Student’s *t* test: *P ≤ 0.05, **P ≤ 0.01. All error bars show SEM. Scale bars in all micrographs are 50 μm. All experiments performed using E18.5 embryos.

To determine the effect of GATA6 loss on ileal epithelial development, we examined epithelial architecture in ileum of control and *Gata6* cKO embryos at E18.5 using H&E staining (Figure 6A). We observed that villi in GATA6-deficient ileum were 26% shorter compared with controls (Figure 6A). Villus length is also decreased when GATA6 is deleted within the ileum of adult mice using a tamoxifen-inducible *Villin-Cre* driver [2]. Because villus length was changed, we compared epithelial cell number in control and mutant tissue by staining with the pan-epithelial cell marker HNF4A and counting HNF4A+ cells per section. As expected and agreeing with Beuling *et al.*[2], we found a 1.7-fold decrease in epithelial cells in GATA6-deficient ileum compared with control ileum (Figure 6B). In contrast to the jejunum, in which loss of GATA4 affects villus length after birth [1] and loss of GATA6 fails to affect villus length [2], loss of GATA6 in the ileum affects villus length during development.

To assess the enterocyte population in GATA6 mutant ileum, we stained for brush border AP activity. We detected robust AP activity in control ileum whereas AP activity was very low or undetectable in GATA6-deficient ileum (Figure 6C). This difference may reflect a decrease in enterocytes in mutant ileum or a shift in the identity of ileal enterocytes toward that of colonocytes as AP activity is normally lower in the colon compared with the small intestine [18]. A similar shift toward colon gene expression was observed when GATA6 is eliminated from the adult ileum [2]. If AP activity changes reflect decreased enterocyte number, we expected to see decreased enterocyte marker expression and no induction of colon markers. If AP activity reflects an identity change, we expected to see reduced enterocyte marker expression and induced colon marker expression. We observed the latter. Expression of the small intestine markers *Fabp2* and *Apoa4* was lost and expression of the colonic marker *Car1* was induced in GATA6 mutants (Figure 6D). Moreover, expression of the intestinal alkaline phosphatase gene (*Alpi)* was lower in mutants compared with controls (Additional file 3). Taken together, these data suggest that enterocyte cell identity is shifted toward the colon in GATA6-deficient ileum.

**Figure 7:**
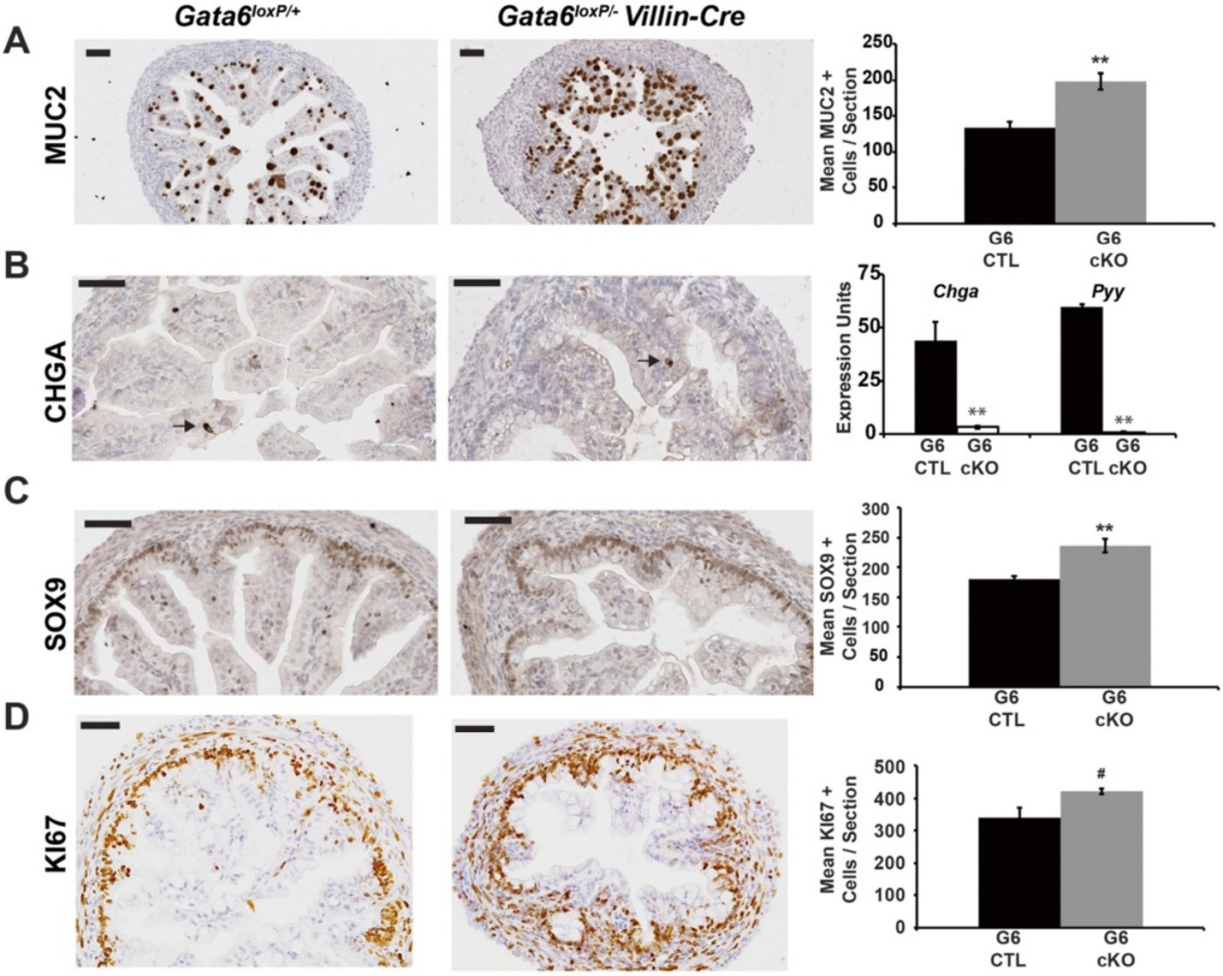
**Loss of GATA6 from the ileal epithelium alters secretory and proliferative cell populations. (A)** Goblet cells, identified by MUCIN 2 (MUC2) IHC, were increased in *Gata6* cKO ileum compared with control tissue (G6 CTL, *Gata6* ^*loxP/+*^, 134 ± 8 MUC2+ cells per section; G6 cKO, *Gata6* ^*loxP/-*^ *Villin-Cre*, 198 ± 11 MUC2+ cells per section; n = 3 embryos/genotype, 3–4 sections/embryo). **(B)** Enteroendocrine cells, stained with Chromogranin A (CHGA), were present in both *Gata6* cKO and control ileum. qRT-PCR was used to determine the abundance of the pan-enteroendocrine cell marker *Chga* and the distal enteroendocrine cell marker *Pyy.* Expression of both was severely decreased in GATA6 mutant ileum compared with control ileum. Epithelial cells from three control (*Gata6* ^*loxP/+*^ *)* and four mutant ileums (*Gata6* ^*loxP/-*^ *Villin-Cre)* were assayed at least three times. *Gapdh* was used for normalization. **(C)** SOX9+ cells were increased in GATA6 mutant ileum compared with control ileum (G6 CTL, *Gata6* ^*loxP/+*^, 181 ± 4 SOX9+ cells/section; G6 cKO, *Gata6* ^*loxP/-*^ *Villin-Cre*, 236 ± 11 SOX9+ cells/section; n = 3 embryos/genotype, 3 sections/embryo). **(D)** KI67+ cells were increased in GATA6 mutant ileum compared with control ileum (G6 CTL, *Gata6* ^*loxP/+*^, 340 ± 30 KI67+ cells/section; G6 cKO, *Gata6* ^*loxP/-*^ *Villin-Cre*, 421 ± 8 KI67+ cells/section; n = 3 embryos/genotype, 5–6 sections/embryo). All *P*-values were determined by two-sample Student’s *t* test: *P ≤ 0.05, **P ≤ 0.01, ^#^ *P* = 0.059. All error bars show SEM. Scale bars in all micrographs are 50 μm. All experiments performed using E18.5 embryos.

To examine goblet cells in mutants, we stained for MUC2 and observed a 1.5-fold increase in goblet cells in GATA6 mutants compared with controls (Figure 7A). Goblet cells are also increased in the ileum of adult *Gata6* cKOs although the increase is limited to the crypt, which does not form until after birth [2]. CHGA IHC identified enteroendocrine cells both in control and GATA6 mutant ileum (Figure 7B). We used *ChgA* qRT-PCR as a measure of enteroendocrine cell number and found decreased *ChgA* expression in GATA6-deficient ileal epithelium compared with control epithelium (Figure 7B). The distal enteroendocrine cell marker *Pyy* was also decreased in GATA6-deficient ileum (Figure 7B). Together, these data suggest that loss of GATA6 in the ileum during development results in fewer enteroendocrine cells. This agrees with the findings of Beuling *et al.*[2] who showed decreased enteroendocrine cells in GATA6-deficient adult ileum. As GATA6 is expressed in enteroendocrine cells (Additional file 2), this may reflect a cell autonomous GATA6 phenotype.

Finally, we examined the intervillus region in control and GATA6 mutant ileum by quantifying SOX9+ cell number (Figure 7C). We found a 1.3-fold increase in SOX9+ cells in GATA6-deficient ileum compared with control ileum suggesting that loss of GATA6 during ileal development causes an expansion of the intervillus region. As the intervillus region contains the proliferative progenitor population, we determined the number of proliferative cells in control and GATA6 mutant ileum by quantifying KI67+ cells (Figure 7D). As predicted by SOX9 staining, we found a slight increase (1.2-fold) in KI67+ cells in GATA6-deficient ileum compared with control ileum. It is somewhat unexpected to find an increase in proliferative cells, yet fewer epithelial cells, in GATA6 mutant ileum. We examined cell death with cleaved caspase 3 IHC and observed only a few positive cells per section in both control and mutant ileum suggesting that increased cell death is not the reason for this disparity (Additional file 4). One alternative explanation is that increased proliferation occurs as a secondary consequence to compensate for epithelial cell loss and decreased villus length. We observed a similar effect when E-cadherin is deleted from the developing small intestine [19]. On the other hand, if this result reflects a direct effect of GATA6 loss, it would suggest that proliferation in the developing intestine is sensitive to total GATA level as we found no change in proliferation in either GATA4 or GATA6 mutant jejunum. When both GATA4 and GATA6 are deleted in the developing jejunum, however, proliferation is increased [12]. Finally, elimination of GATA6 from the adult ileum results in fewer proliferative cells in mature crypts [2]. This suggests that the requirement for GATA6 with respect to proliferation varies over time in the ileum.

## Conclusions

This study investigated the impact of loss of either GATA4 or GATA6 within the intestinal epithelium during development. We found that intestinal development was generally normal in GATA4 and GATA6 mutants. Phenotypes observed in embryonic GATA4 or GATA6 mutant small intestine paralleled those identified in adult small intestine lacking either GATA4 or GATA6. The effect of loss of GATA6 in the developing ileum was greater than the effect of loss of either GATA4 or GATA6 in the developing jejunum. As the jejunum co-expresses GATA4 and GATA6 whereas the ileum expresses only GATA6, we conclude that overlapping GATA4 and GATA6 function in the jejunum lessens the effect of loss of either factor alone in this tissue. Although our data demonstrate redundancy between GATA4 and GATA6 function within the developing jejunum, examination of single knockouts suggests that GATA4 likely functions independently of GATA6 in the jejunum to regulate jejunal versus ileal enterocyte identity. Moreover, GATA6 likely plays a direct role in regulating enteroendocrine cell differentiation whereas GATA4 affects enteroendocrine cell development indirectly.

## Methods

### Animals

CD-1 mice (Charles River Laboratories, Wilmington, MA) were used to determine expression of GATA4 in embryonic small intestine. The *Rosa26* conditional reporter strain *Gt(ROSA)26Sor*^*tm1Sor*^*(Rosa26R)* was used to determine Cre activity [16]. *Gata4*^*tm1.1Sad*^*(Gata4*^*loxP*^), *Gata4*^*tmo1Eno*^ (*Gata4*^*−*^), *Gata6*^*tm2.1Sad*^ (*Gata6*^*loxP*^), *Gata6*^*tm2.2Sad*^ (*Gata6*^*−*^), and Tg(*Vil-cre*)997Gum (*Villin-Cre*) mice were used to generate *Gata4* cKO (*Gata4*^*loxP/-*^*Villin-Cre)* and *Gata6* cKO (*Gata6*^*loxP/-*^*Villin-Cre)* embryos [10, 13–15]. Control genotypes were either *Gata4*^*loxP/+*^ or *Gata6*^*loxP/+*^. Embryos were obtained by timed mating with noon of the day of a vaginal plug considered as embryonic day (E) 0.5. Genotypes were determined by PCR using primers previously described [1, 12]. The Medical College of Wisconsin’s Animal Care Committee approved all animal procedures.

### Intestinal epithelial cell isolation

Small intestine (jejunum or ileum) was isolated from E18.5 embryos, cut longitudinally, and incubated in cell dissociation buffer (BD Biosciences, San Jose, CA) for at least 6 hours at 4°C with shaking [20, 21]. Mesenchymal tissue was removed, and epithelial cells were used to obtain total RNA for qRT-PCR experiments. Jejunal samples excluded epithelial cells from the distal 1–2 cm of the embryonic small intestine. Ileal samples contained epithelial cells from the distal 0.5-1.0 cm of the embryonic small intestine.

### Reverse transcription polymerase chain reaction

DNase treated RNA was used to generate cDNA from isolated epithelial cells as previously described [19, 22]. Additional file 5 contains a table listing the TaqMan assays identifiers used for qRT-PCR (Life Technologies, Carlsbad, CA). *Gapdh* was used as a normalization control. Expression units for each target represent (2^-ΔCq^)*1000. Multiplying by a factor of 1000 adjusted most expression unit values to >1. For all assays, gene expression was measured at least three times using cDNA generated from 3 control (*Gata4*^*loxP/+*^, jejunal assays; *Gata6*^*loxP/+*^, ileal assays) and 3–4 experimental (*Gata4*^*loxP/-*^*Villin-Cre,* jejunal assays*; Gata6*^*loxP/-*^*Villin-Cre* jejunal and ileal assays) E18.5 embryos. *P*-values were determined by a two-sample Student’s *t* test. Error bars represent standard error of the mean (SEM).

### Histochemistry and immunohistochemistry

Histochemistry and immunohistochemistry were performed using previously described methods [19, 22]. Jejunal tissue was harvested from the midpoint of the small intestine. The distal 0.5- 1.0 cm of the embryonic small intestine was considered ileum for these assays. To assay β-galactosidase activity in tissue, embryonic intestine was dissected and fixed in 4% paraformaldehyde for 15 minutes at room temperature. Intestines were washed and incubated overnight in X-gal (5-bromo-4-chloro-3-indolyl-β-D-galactoside) stain as previously described [23]. Antibodies used for IHC are listed in Additional file 6.

## Electronic supplementary material

Additional file 1: **qRT-PCR for** ***Gata4*** **and** ***Gata6*** **in conditional knockout intestinal tissue.** qRT-PCR was used to determine the abundance of *Gata4* and *Gata6* mRNA in control and mutant jejunum and ileum*.* For jejunal assays, epithelial cells from three control (*Gata4* ^*loxP/+*^ *)* and three mutant jejunums (*Gata4* ^*loxP/-*^ *Villin-Cre* and *Gata6* ^*loxP/-*^ *Villin-Cre)* were assayed at least three times. For ileal assays, epithelial cells from three control (*Gata6* ^*loxP/+*^ *)* and four mutant ileums (*Gata6* ^*loxP/-*^ *Villin-Cre)* were assayed at least three times. *Gapdh* was used for normalization. All *P*-values were determined by two-sample Student’s *t* test: *P ≤ 0.05. Error bars show SEM. All tissue harvested was from E18.5 embryos. (JPEG 196 KB)

Additional file 2: **Immunohistochemical staining for GATA6 and Chromogranin A in the mouse small intestine.** E18.5 wild-type tissue was co-stained for GATA6 (brown nuclear stain) and Chromogranin A (CHGA; brown cytoplasmic stain) and counterstained with hematoxylin. Higher magnification images of the boxed regions are shown at right. Stars indicate GATA6+, CHGA+ enteroendocrine cells. Scale bar, 50 μm. (JPEG 2 MB)

Additional file 3: **Semi-quantitative RT-PCR to determine the expression level of the** ***intestinal alkaline phosphatase*** **gene (** ***Alpi*** **) in control (** ***Gata6*** ^***loxP/+***^ ***)*** **and** ***Gata6*** **cKO (** ***Gata6*** ^***loxP/-***^ ***Villin-Cre)*** **ileal epithelium.** Epithelial cells from three controls and four mutants were assayed. PCR was performed including ^α32^P-dATP. To quantify fold change, a Phosphorimager (Molecular Dynamics) was used. Samples were normalized to the level of *Polr2a* expression. Data shown are representative of two experiments. Primer sequences are included in Additional file 5. (JPEG 136 KB)

Additional file 4: **Cleaved caspase 3 immunohistochemistry in control and GATA6 mutant ileum.** E18.5 control (*Gata6* ^*lox/+*^ *)* and GATA6 mutant (*Gata6* ^*loxP/-*^ *Villin-Cre)* ileum were stained for cleaved caspase 3 (brown nuclear stain). Very few cleaved caspase 3 positive epithelial cells were observed in ileum of either genotype (n = 3 embryos/genotype; 3–4 sections/embryo examined). Higher magnification images of the boxed regions are shown as insets. Arrows indicate cleaved caspase 3 positive cells. Staining from adult control tissue is shown as a positive control for antibody staining. Several cleaved caspase positive cells are present at the villus tip. (JPEG 1 MB)

Additional file 5: **TaqMan assays used for qRT-PCR.** List of the identifiers for TaqMan primer/probe sets used in qRT-PCR analyses and primers used in semi-quantitative RT-PCR analyses. (PDF 59 KB)

Additional file 6: **Antibodies.** List of the antibodies used in study. Dilutions used, manufacturer, and catalog numbers are provided. (PDF 65 KB)
