# Supplementary figures and images for: Characterization of the developing small intestine in the absence of either GATA4 or GATA6
Source: BMC Res Notes. 2014 Dec 11;7:902. doi: 10.1186/1756-0500-7-902 (PMC4307969; doi:10.1186/1756-0500-7-902)

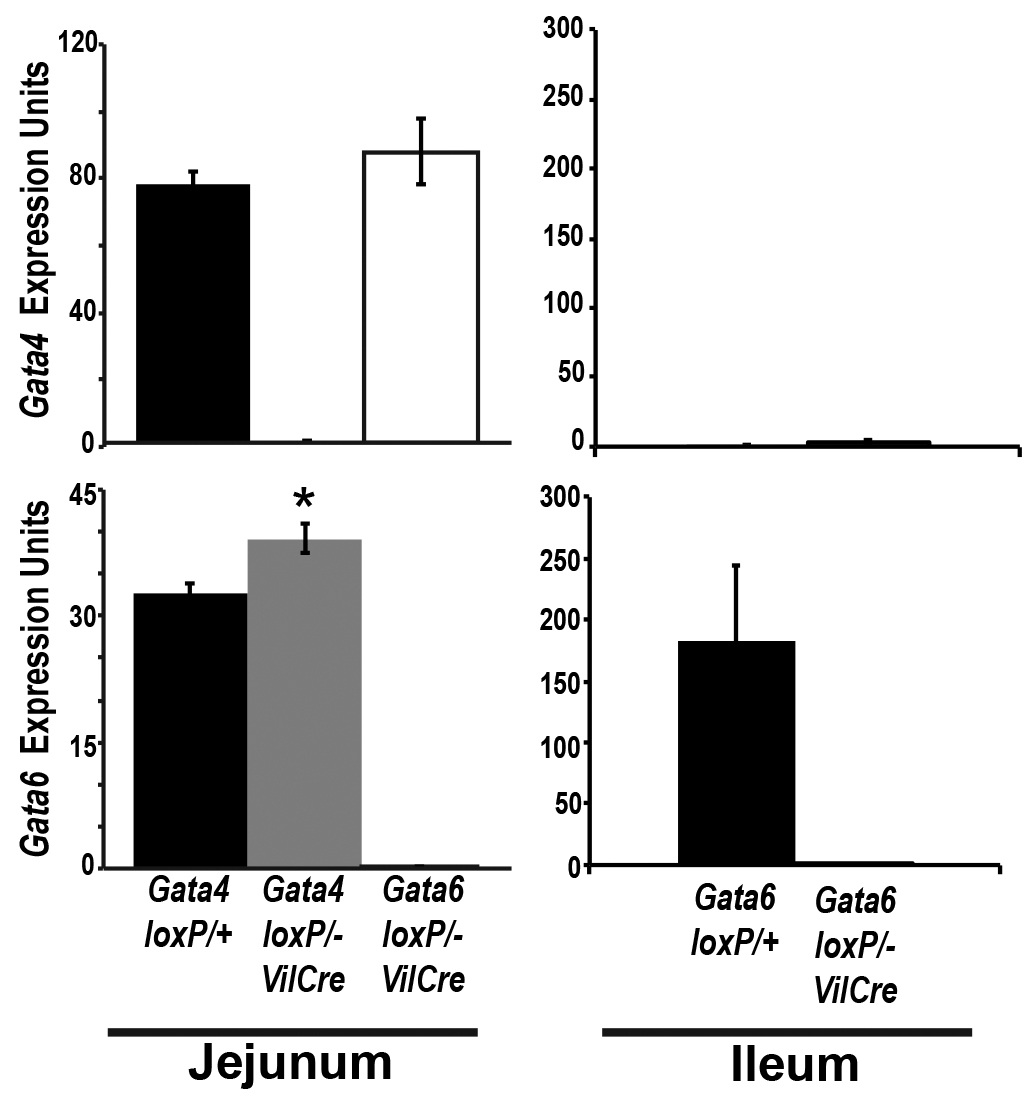

Supplement: Supplementary file 1 — Additional file 1: qRT-PCR for Gata4 and Gata6 in conditional knockout intestinal tissue. qRT-PCR was used to determine the abundance of Gata4 and Gata6 mRNA in control and mutant jejunum and ileum. For jejunal assays, epithelial cells from three control (Gata4 loxP/+ ) and three mutant jejunums (Gata4 loxP/- Villin-Cre and Gata6 loxP/- Villin-Cre) were assayed at least three times. For ileal assays, epithelial cells from three control (Gata6 loxP/+ ) and four mutant ileums (Gata6 loxP/- Villin-Cre) were assayed at least three times. Gapdh was used for normalization. All P-values were determined by two-sample Student’s t test: *P ≤ 0.05. Error bars show SEM. All tissue harvested was from E18.5 embryos. (JPEG 196 KB) [file 13104_2014_3474_MOESM1_ESM.jpeg]

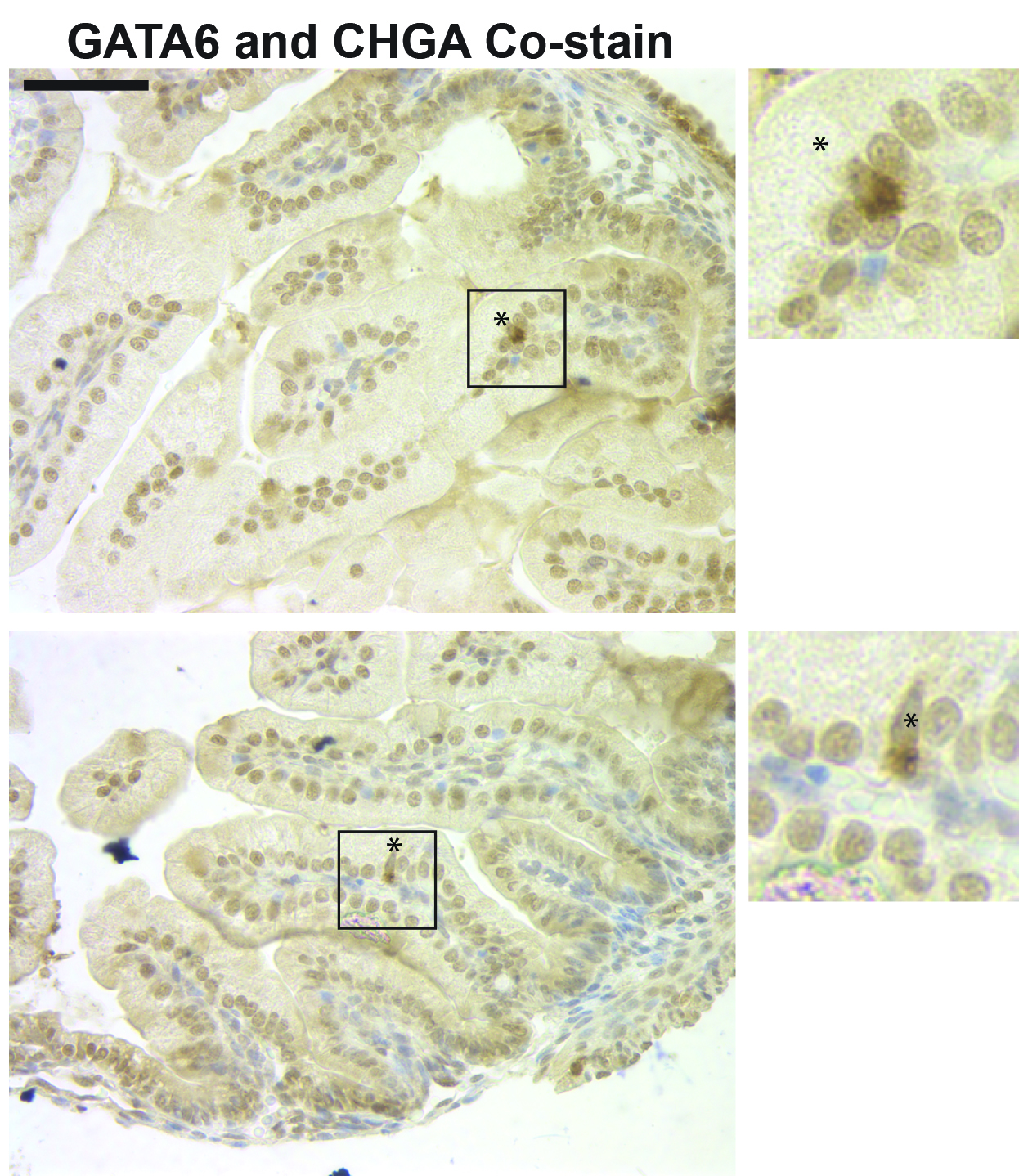

Supplement: Supplementary file 2 — Additional file 2: Immunohistochemical staining for GATA6 and Chromogranin A in the mouse small intestine. E18.5 wild-type tissue was co-stained for GATA6 (brown nuclear stain) and Chromogranin A (CHGA; brown cytoplasmic stain) and counterstained with hematoxylin. Higher magnification images of the boxed regions are shown at right. Stars indicate GATA6+, CHGA+ enteroendocrine cells. Scale bar, 50 μm. (JPEG 2 MB) [file 13104_2014_3474_MOESM2_ESM.jpeg]

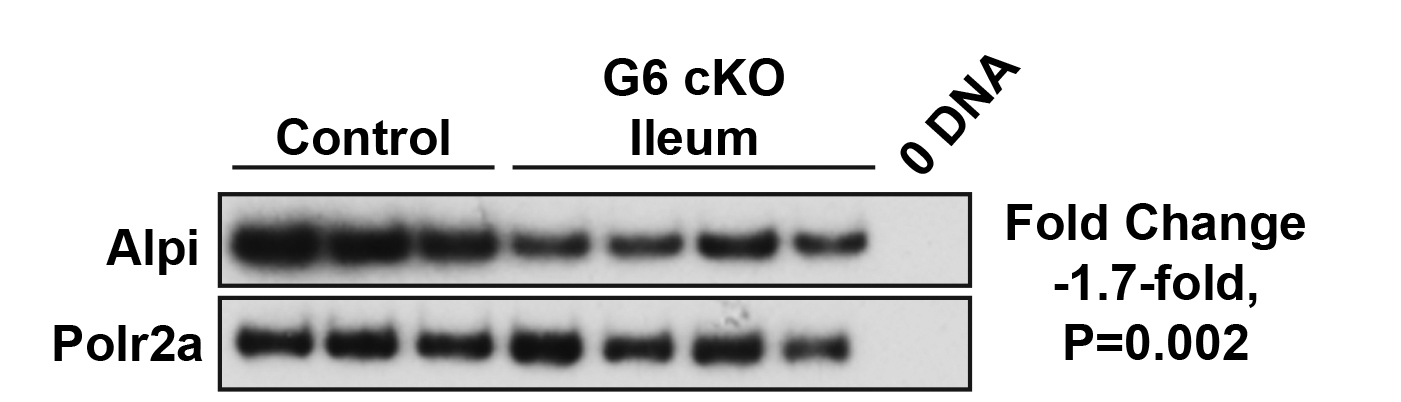

Supplement: Supplementary file 3 — Additional file 3: Semi-quantitative RT-PCR to determine the expression level of the intestinal alkaline phosphatase gene ( Alpi ) in control ( Gata6 loxP/+ ) and Gata6 cKO ( Gata6 loxP/- Villin-Cre) ileal epithelium. Epithelial cells from three controls and four mutants were assayed. PCR was performed including α32P-dATP. To quantify fold change, a Phosphorimager (Molecular Dynamics) was used. Samples were normalized to the level of Polr2a expression. Data shown are representative of two experiments. Primer sequences are included in Additional file 5. (JPEG 136 KB) [file 13104_2014_3474_MOESM3_ESM.jpeg]

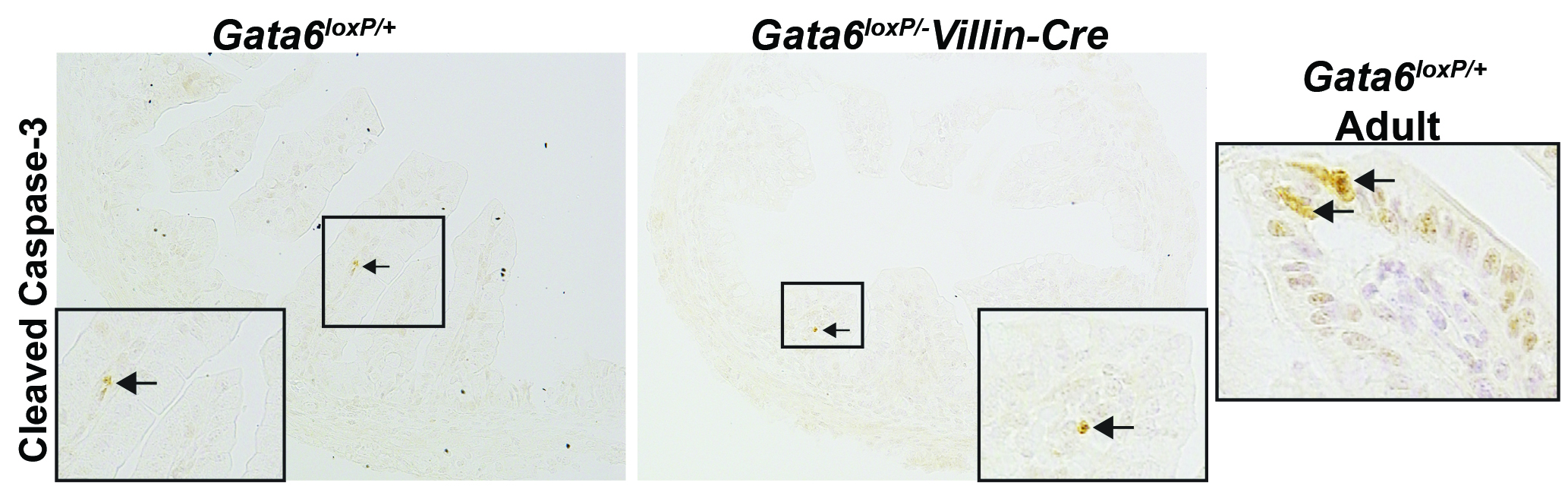

Supplement: Supplementary file 4 — Additional file 4: Cleaved caspase 3 immunohistochemistry in control and GATA6 mutant ileum. E18.5 control (Gata6 lox/+ ) and GATA6 mutant (Gata6 loxP/- Villin-Cre) ileum were stained for cleaved caspase 3 (brown nuclear stain). Very few cleaved caspase 3 positive epithelial cells were observed in ileum of either genotype (n = 3 embryos/genotype; 3–4 sections/embryo examined). Higher magnification images of the boxed regions are shown as insets. Arrows indicate cleaved caspase 3 positive cells. Staining from adult control tissue is shown as a positive control for antibody staining. Several cleaved caspase positive cells are present at the villus tip. (JPEG 1 MB) [file 13104_2014_3474_MOESM4_ESM.jpeg]
