# Supplementary material for: Characterization of the developing small intestine in the absence of either GATA4 or GATA6
Source: BMC Res Notes. 2014 Dec 11;7:902. doi: 10.1186/1756-0500-7-902 (PMC4307969; doi:10.1186/1756-0500-7-902)
Supplement: Supplementary file 5 — Additional file 5: TaqMan assays used for qRT-PCR. List of the identifiers for TaqMan primer/probe sets used in qRT-PCR analyses and primers used in semi-quantitative RT-PCR analyses. (PDF 59 KB) [file 13104_2014_3474_MOESM5_ESM.pdf]

TaqMan probe sets and semi-quantitative radioactive primer sets used for qRT-PCR

Gene	TaqMan ID	Gene	TaqMan ID
Abcg5	Mm00433937_m1	*Fgf15*	Mm00433278_m1
Abcg8	Mm00433937_m1	*Gapdh*	Mm99999915_g1
Apoa4	Mm00431814_m1	*Gata4*	Mm00484689_m1
Apoc2	Mm00433937_m1	*Gata6*	Mm00802632_m1
Apoc3	Mm00433937_m1	*Gip*	Mm00433601_m1
Car1	Mm00486717_m1	*Lct*	Mm01285112_m1
Cck	Mm00446170_m1	*Ngn3*	Mm00437606_s1
Chga	Mm00514341_m1	*Pyy*	Mm00520716_g1
Cldn8	Mm00516972_s1	*Slc2a2*	Mm00433937_m1
Fabp1	Mm00444340_m1	*Slc2a5*	Mm00433937_m1
Fabp2	Mm00433188_m1	*Slc5a11*	Mm00433937_m1
Fabp6	Mm00434316_m1	*Slc10a2*	Mm00488258_m1

Gene	Forward Primer	Reverse Primer	PCR product size (bp)
Alpi	aatgctagtttttgcaccccaactt	ccactccagaggaagtcagttgaaa	163
Polr2a	ctgatgcgggtgctgagtgagaagg	gcggttgaccccatgacgagtg	237
