# Supplementary material for: Characterization of the developing small intestine in the absence of either GATA4 or GATA6
Source: BMC Res Notes. 2014 Dec 11;7:902. doi: 10.1186/1756-0500-7-902 (PMC4307969; doi:10.1186/1756-0500-7-902)
Supplement: Supplementary file 6 — Additional file 6: Antibodies. List of the antibodies used in study. Dilutions used, manufacturer, and catalog numbers are provided. (PDF 65 KB) [file 13104_2014_3474_MOESM6_ESM.pdf]

Antibodies used for Immunohistochemistry

Antibody	Dilution	Manufacturer	Catalog Number
GATA4 C-20 (goat polyclonal)	1:250	Santa Cruz Biotechnology, Santa Cruz, CA	sc-1237
GATA6 (rabbit polyclonal)	1:4000	Gift of Xiang-Xi (Mike) Xu, Miller School of Medicine, University of Miami, Miami, FL	N/A
CHROMOGRANIN A (rabbit polyclonal)	1:4000	ImmunoStar, Hudson, WI	20085
Cleaved Caspase-3 (Asp 175) (rabbit polyclonal)	1:250	Cell Signaling, Danvers, MA	9661
HNF4a (goat polyclonal)	1:500	Santa Cruz Biotechnology, Santa Cruz, CA	sc-6556
MUCIN 2 (rabbit polyclonal)	1:250	Santa Cruz Biotechnology, Santa Cruz, CA	sc-15334
KI67 (rabbit polyclonal)	1:100 or 1:200	Biocare Medical, Concord, CA	CRM325B
SOX9 (guinea pig polyclonal)	1:1500	Gift of Dr. Vivian Lee, Medical College of Wisconsin, Milwaukee, WI	N/A
Biotinylated goat anti-rabbit (H+L) IgG	15 μl/ml	Vector Labs, Burlingame, CA	BA-1000
Biotinylated goat anti-guinea pig IgG	15 μl/ml	Vector Labs, Burlingame, CA	BA-7000
Biotinylated rabbit anti-goat IgG	15 μl/ml	Vector Labs, Burlingame, CA	BA-5000
